# Preoperative Computed Tomography is Associated with Reduced In-Hospital Complications in Aortic Valve Surgery

**DOI:** 10.1055/a-2708-3100

**Published:** 2025-10-24

**Authors:** Liliane Zillner, Julian Heidtmann, Markus Mach, Richard Nolz, Christian Loewe, Alfred Kocher, Daniel Zimpfer, Martin Andreas

**Affiliations:** 1 Department of Cardiac Surgery, Medical University of Vienna, Vienna, Austria; 2 Department of Biomedical Imaging and Image-Guided Therapy, Medical University of Vienna, Vienna, Austria

**Keywords:** aortic valve replacement, perioperative stroke, computed chest tomography, in-hospital complications, full aortic computed tomography

## Abstract

**Objective:**

To assess the efficacy of preoperative full aortic computed tomography (CT) to reduce complications during surgical aortic valve replacement (SAVR).

**Methods:**

A single-center retrospective study examined all SAVR procedures from 2013 to 2015, comparing outcomes between surgeries planned with CT and those without. The study assessed how CT imaging adapted surgical methods, including cannulation and the possibility of switching from SAVR to interventional therapy. The analysis primarily focused on the occurrence of in-hospital complications.

**Results:**

Out of 359 patients analyzed, those who received presurgical CT ( *n* = 305, complications = 53; 17%; EuroSCORE = 1.8) had fewer in-hospital complications compared with the non-CT group ( *n* = 54, complications = 17; 32%; EuroSCORE = 1.8), with a statistically significant difference ( *p* = 0.016). Patients in the CT group had a 15% absolute risk reduction and a number needed to treat of 7 to avoid one in-hospital complication.

**Conclusion:**

CT is associated with reduced in-hospital complications in SAVR patients and could enhance patient outcomes when used in preoperative planning. This supports the recommendation for incorporating CT into routine preoperative assessment to enable personalized surgical strategies, potentially including a shift to transcatheter treatments when indicated.

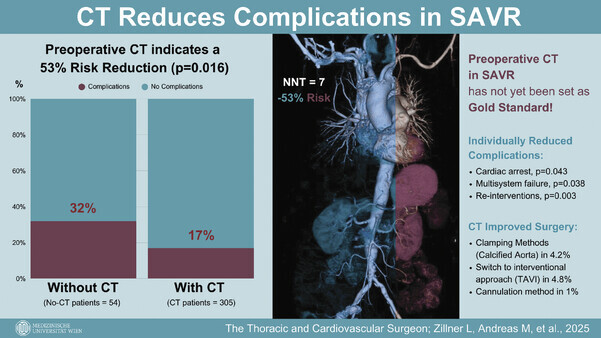

## Introduction

Full aortic computed tomography (CT) performed in preparation for surgical aortic valve replacement (SAVR) can serve as a critical diagnostic tool as it provides detailed images of the heart, aorta, and surrounding structures. Crucial insights as the extent of calcification are gained, and coexisting conditions can be diagnosed, potentially preventing stroke and other complications. 1 2 3 4

Unlike in transcatheter aortic valve implantation (TAVI), where preinterventional CT is mandatory for assessing valve and vascular calcification and feasibility of vascular access, the use of CT prior to SAVR is often reserved for exceptional cases. While echocardiography alone remains the gold standard for diagnosing aortic stenosis (AS) prior to surgery, CT may provide additional information regarding valve calcification (Agatston score). 5 6

An additional CT-based roadmap of the heart's anatomy might influence important surgical steps like the placement of the aortic clamp, choosing the ideal access route, and cannulation side (minimally invasive or regular approach) or even change the whole therapeutic journey by leading the heart team's decision toward a percutaneous intervention (TAVI). 7 8

By contrast, conventionally used echocardiography alone is safe but examiner dependent and limited. While transthoracic echocardiography (TTE) is essential in diagnosis and follow-up by evaluating valve function, it can only play a minor role in surgical planning. 9 10 Perioperatively used transesophageal echocardiography has limited opportunities to evaluate the arteriosclerotic status of the aortic arch due to anatomical restrictions like the tracheal column. 11 12 13 After sternotomy, epiaortic ultrasonography may be applied and can impact cannulation and clamping but cannot influence previous surgical decision-making and planning. 14 15 16

We hypothesized that complications can be significantly reduced by routine preoperative CT prior to SAVR. The aim of the study was to unveil CT's potential to guide the surgeon and the entire heart team toward a safer intervention by impacting surgical strategies and patient selection, eventually leading to a decrease in complications.

## Materials and Methods

### Study Design and Population

The Medical University of Vienna's Ethics Committee granted approval for this study (reference no. 1213/2014), which was conducted as a single-center retrospective analysis. Our review included 359 patients who underwent a primary, planned, and isolated aortic valve replacement (AVR) at the Medical University of Vienna from 2013 to 2015. These patients were either evaluated with preinterventional full aortic CT or not. A total of 305 patients who received routine preoperative CT and 54 who did not undergo preinterventional CT were enrolled in the study.

We focused on the incidence of in-hospital complications and mortality as the primary outcome endpoints. The complications analyzed in our study included perioperative myocardial infarction, anticoagulation complications, postoperative stroke within 72 hours, and other neurological complications categorized as minor or major. Additionally, continuous coma lasting over 24 hours, prolonged ventilation, pulmonary embolism, iliac femoral dissection, acute limb ischemia, tamponade, multisystem failure, aortic dissection, and the need for extracorporeal membrane oxygenation support were assessed. The study also examined reinterventions due to reasons like bleeding, tamponade, valve dysfunction, and sternal reintervention. Cardiac arrest, triggered by postoperative arrhythmias, ischemia, bleeding, and other complications, as well as in-hospital mortality, were also analyzed.

### Data Usage

Concentrating exclusively on data collected up to the year 2015, this research examines an era characterized by a consistent surgical environment prior to the rise of transcatheter therapies, which enhances the precision of our examination into the direct effects of CT imaging on surgical methods within that timeframe. Additionally, it enabled the assessment of long-term mortality rates. From 2015 onward, there have been notable evolutions in both the protocols and the applied methods within the realm of AS treatment. These include the broader application criteria for TAVI, prompted by recent guidelines, and advancements in both imaging and surgical techniques, all of which have significantly impacted the strategic planning of valve surgeries. 17 18

### Statistical Analysis

In this study, 359 patients who underwent aortic valve surgery were analyzed and categorized into two cohorts: those who had undergone preinterventional full aortic CT ( *n* = 305) and those who had not ( *n* = 54). The Kolmogorov–Smirnov test revealed that data were not normally distributed regarding continuous variables. Baseline parameters, risk analysis, and complication rates for each group were evaluated using the Mann–Whitney *U* test for continuous variables and the chi-squared (χ ^2^ ) test for categorical variables. We considered a *p* -value of <0.05 to be statistically significant. The 2021 version of SPSS was used for analyses. Analysis revealed that the groups were remarkably homogeneous, with the most baseline characteristics showing negligible differences, thereby constituting a valid and comparable cohort.

Because this was a retrospective, nonrandomized study, patients with and without CT could differ in baseline surgical risk. To reduce this bias, we applied a propensity score weighting approach. The propensity score was estimated using the logarithm of the EuroSCORE I (logEuroScoreI) as predictor, and average treatment effect on the treated (ATT) weighting was performed, thereby mimicking randomization with respect to surgical risk.

## Results

### Baseline and Procedural Characteristics

In this study, 359 patients who underwent isolated AVR surgery from 2013 to 2015 at a single institution were analyzed. Baseline characteristics of both cohorts are detailed in Table 1 . The homogeneous study cohorts presented a median EuroSCORE of 1.8 for patients who had undergone CT and those who had not. The use of CT increased steadily over time. In 2013, 79.1% of patients received CT, rising to 86.9% in 2014 and 88.5% in 2015.

**Table 1:** Baseline data

Demographics (total = 359)	CT ( *n* = 305)	No CT ( *n* = 54)	*p* -Value
Metric values	Md (interquartile range)		
LogEuroSCORE I	5.69 (6.61)	6.19 (10.93)	0.52
EuroSCORE II	1.8 (2)	1.8 (2.45)	0.70
Age (y)	71 (14)	69.5 (17.5)	0.30
Weight (kg)	80 (20)	83.5 (27)	0.68
BMI (kg/m ^2^ )	27.99 (6.4)	27.27 (6.8)	0.94
Creatinine (mg/dL)	0.94 (0.31)	1 (0.39)	0.52
EF (%)	60 (10)	55 (11)	**0.034**
Gradient (mm Hg)	50 (24.8)	46 (24)	0.18
Dichotomous values	*n* (%)		
Male	169 (55.4)	28 (51.8)	0.63
Female	136 (44.6)	26 (48)	0.63
Octogenarian	43 (14)	8 (14.8)	0.89
Smoker	88 (28.9)	16 (30)	0.91
Current smoker	25 (8.2)	5 (9.3)	0.80
Diabetes	77 (25.3)	12 (22.2)	0.64
Dyslipidemia	195 (64)	36 (66.6)	0.70
Renal failure	41 (13.4)	8 (14.8)	0.79
Dialysis	10 (3.3)	0 (0)	0.18
Hypertension	259 (84.9)	47 (87)	0.69
CVA	26 (8.5)	6 (11.1)	0.54
TIA (minor event)	2 (0.7)	2 (3.7)	0.14
Stroke (major event)	24 (7.9)	4 (7.4)	0.14
Endocarditis	16 (5.3)	7 (13)	**0.033**
COPD Grade 1	52 (17)	15 (27.8)	0.25
COPD Grade 2	20 (6.6)	2 (3.7)	0.25
COPD Grade 3	8 (2.6)	2 (3.7)	0.25
PVD	23 (7.5)	5 (9.3)	0.67
CVD	39 (12.8)	9 (16.7)	0.44
Prev. CV interven.	56 (18.4)	8 (14.8)	0.53
Prev. CABG	4 (1.3)	2 (3.7)	0.21
Prev. valve repair	29 (9.5)	2 (3.7)	0.16
Previous PCI (other)	19 (6.2)	2 (5.7)	0.47
MI	11 (3.6)	4 (7.4)	0.20
Angina			
Stable angina	52 (17)	6 (11.1)	0.37
Unstable angina	4 (1.3)	0 (0)	0.37
Arrhythmia	57 (18.7)	15 (27.8)	0.12
Atrial fibrillation	47 (15.4)	9 (16.7)	0.12
AV block	5 (1.6)	9 (16.7)	0.12
NYHA I	20 (6.6)	3 (5.6)	0.43
NYHA II	98 (32.1)	12 (22.2)	0.43
NYHA III	173 (56.7)	35 (64.8)	0.43
NYHA IV	14 (4.6)	4 (7.4)	0.43
Neurological Dysf.	6 (2)	3 (5.6)	0.12
AP therapy	179 (59)	35 (64.8)	0.40
Single AP therapy	127 (41.6)	27 (44.4)	0.25
Dual AP therapy	5 (1.6)	1 (1.9)	0.91
Marcumar/heparin	57 (18.7)	11 (20.4)	0.78
Lipid Low. Med.	159 (52)	22 (37)	0.12
ADP inhibitors	10 (3.3)	3 (7.5)	0.41
Coronary VD I	31 (10.2)	2 (5.6)	0.33
Coronary VD II	9 (3)	1 (1.9)	0.33
Coronary VD III	5 (1.6)	2 (3.7)	0.33
Elective (status)	260 (85.2)	43 (80)	**0.001**
Urgent (status)	41 (13.4)	5 (9.3)	**0.001**
Emergency (status)	4 (1.3)	6 (11.1)	**0.001**

Abbreviations: AP therapy, antiplatelet therapy; AV block, atrioventricular block; BMI, body mass index; CABG, coronary artery bypass grafting; CAD, coronary artery disease; Coronary VD I–III, extent of coronary vessel disease; CVA, cerebrovascular accident; CVD, cerebrovascular disease; DAPT, dual antiplatelet therapy; EF, ejection fraction; EuroSCORE II, European System for Cardiac Operative Risk Evaluation II; lipid-lowering med., lipid-lowering medication; LogEuroSCORE I, logistic EuroSCORE I; MI, myocardial infarction; Neurological Dysf., neurological dysfunction; NYHA, New York Heart Association Classification; PCI, percutaneous coronary intervention; Prev. CV Interven., previous cardiovascular intervention; PVD, peripheral vascular disease; TIA, transient ischemic attack.

Notes: Metric values are presented as median (Md) and interquartile range (IQR). Dichotomous values are shown as absolute numbers with percentages in parentheses.

### Outcomes

In-hospital complications were observed in 53 patients (17%) within the CT cohort and 17 patients (32%) within the non-CT cohort ( *p* = 0.016), as outlined in Table 2 . Consequently, patients who underwent AVR with preoperative CT, aimed at enhancing surgical planning and strategy, experienced a 55% reduction in the risk of in-hospital complications. The absolute risk reduction was 15%, and conversely, the number needed to treat was 7. A total of 70 complications were indicated. 30-day mortality was 2.6% with preoperative CT ( *n* = 8, LogEuroSCORE I = 5.69, EuroSCORE II = 1.8) and 3.9% without, *p* = 0.66 ( *n* = 2, LogEuroSCORE I = 6.19, EuroSCORE II = 1.8).

**Table 2:** Risk analysis

Number of patients	Number of events, *n* (%)	Relative risk	Absolute risk reduction (%)	NNT	*p* -Value
	In-hospital complications				
Total (359)	70 (19.50%)				
CT (305)	53 (17.38%)	0.55	15	7	0.016
No CT (54)	17 (31.48%)	Reference			

Abbreviations: NNT, number needed to treat; CT, computed tomography.

Note: In-hospital complications and risk rates in patients with and without presurgical CT.

In our total study cohort, SAVR was conducted through three types of surgical access: sternotomy (53%), hemisternotomy (26%), and thoracotomy (21%). Regarding to access modalities, there was no significant variation between both cohorts and patients with additional CT evaluation showed significance or trends toward fewer complication rates, whether accessing via sternotomy (21.8%, *p* = 0.44), hemisternotomy (11.3%, *p* = 0.59), or thoracotomy (14.5%, *p* = 0.44).

### Propensity Weighting

Prior to adjustment, the logEuroSCORE I differed markedly between groups, with higher mean values in the no-CT group (12.05 ± 15.45) compared with the CT group (8.57 ± 9.46). This corresponded to a standardized mean difference (SMD) of –0.27, indicating relevant imbalance (>0.1). After ATT adjustment, the mean values were 11.36 ± 15.27 (no-CT) versus 10.29 ± 11.47 (CT), with the SMD reduced to –0.08, suggesting adequate balance (<0.1, the conventional threshold indicating well-balanced groups).

In the unadjusted logistic regression, CT was associated with significantly lower odds of postoperative complications (odds ratio [OR] = 0.46, 95% confidence interval [CI]: 0.24–0.87, *p* = 0.018). After ATT adjustment, the effect remained significant but was attenuated (OR = 0.62, 95% CI: 0.42–0.91, *p* = 0.016).

### Complications

In the presurgical CT group, a significant reduction in overall complications was observed, with cardiac arrest ( *p* = 0.043), multisystem failure ( *p* = 0.038), and reinterventions (reinterventions with CT = 50, 16.5%, reinterventions without CT = 18, 24%, *p* = 0.003) identified as independent factors contributing to this decrease. Causes for reinterventions included bleeding (tamponade), pericardial effusion, valve dysfunction, other cardiac causes, as well as noncardiac causes associated to surgery such as hematoma evacuation, sternal stabilization, or treatment of sternal infection.

Since minimally invasive approaches may result in fewer noncardiac complications related to surgery, we confirmed a trend toward fewer complication rates, regardless of access modality as mentioned above. While the effect of CT remained significant ( *p* = 0.029; OR = 0.485), confirming that the reduction in complications associated with CT use is robust, the surgical access type also showed an independent effect on complications ( *p* = 0.050; OR = 0.700).

In multivariate models including CT and urgency status, the impact of CT on complication rates approached significance ( *p* = 0.053; OR = 0.517), suggesting a 48% reduction in complications. As anticipated, the patient's urgency status significantly predicted complications ( *p* = 0.002; OR = 2.200). Furthermore, because urgency status may have triggered the indication for CT in some patients, this factor could have confounded the outcomes and led to an underestimation of the benefits of presurgical CT that our study nonetheless indicates. A breakdown of the analyzed complications is listed in Table 3 .

Importantly, our additional analysis, which excluded patients with infectious endocarditis ( *n* = 336)—considering that the pathogenesis of AS induced by infectious endocarditis widely differs from that caused by atherosclerotic AS—revealed that reinterventions for any cause in association with AVR were also significantly less common in the CT-guided cohort. Specifically, 47 complications (16.3%) were observed in the CT group compared with 15 complications (31.9%) in the non-CT group, *p* = 0.01. Thus, procedures planned by CT exhibited an absolute risk reduction of 15.65% (RR = 51%). Similarly to the results of the complete study population, patients aged 75 and older had significantly fewer in-hospital complications when receiving guidance from preoperative CT (25 vs. 57%; *p* = 0.012; Table 4 ).

**Table 3:** Complications within the study cohort with presurgical full aortic computed tomography and the cohort without computed tomography

Complications	CT ( *n* = 303)		No CT ( *n* = 54)		Total ( *n* = 357)		*p* -Value
	Yes *n* (%)	No *n* (%)	Yes *n* (%)	No *n* (%)	Yes *n* (%)	No *n* (%)	
Total	53 (17.5)	250 (82.5)	17 (23.3)	37 (76.7)	70 (19.6)	287 (80.4)	0.016
Perioperative MI	2 (0.7)	301 (99.3)	0 (2.2)	54 (97.8)	2 (0.6)	355 (99.4)	0.55
Anticoag. Compl.	3 (1)	300 (99)	1 (1.4)	53 (98.6)	4 (1.1)	353 (98.9)	0.58
Post. stroke (72h)	4 (1.3)	299 (98.7)	0 (0)	54 (100)	4 (1.1)	353 (98.9)	0.40
Other Neur. Compl.							
Minor	14 (4.6)		4 (5)		18 (5)		0.38
Major	0 (0)		0 (0)		0 (0)		
Cont. coma (>24h)	2 (0.7)	301 (99.3)	0 (0)	54 (100)	2 (0.6)	355 (99.4)	0.55
Prol. ventilation	17 (5.6)	286 (94.4)	3 (3.9)	51 (96.1)	20 (5.6)	337 (94.4)	1.00
Pulm. embolism	0 (0)	303 (100)	0 (0)	54 (100)	0 (0)	357 (100)	
Iliac Fem. Diss.	2 (0.7)	301 (99.3)	0 (0)	54 (100)	2 (0.6)	355 (99.4)	0.55
Acute Limb. Isch.	0 (0)	303 (100)	0 (0)	54 (100)	0 (0)	357 (100)	
Tamponade	4 (1.3)	299 (98.7)	2 (2.5)	52 (97.5)	6 (0.6)	351 (99.4)	0.21
Multisystem failure	4 (1.3)	299 (97.7)	3 (1.4)	51 (98.6)	7 (2)	350 (98)	**0.038**
Aortic dissection	0 (0)	303 (100)	0 (0)	54 (100)	0 (0)	357 (100)	
ECMO	4 (1.3)	299 (98.7)	2 (2.9)	52 (97.1)	6 (1.7)	356 (98.3)	0.21
Reintervention (any cause) a	50 (16.5)	253 (83.5)	18 (24)	36 (76)	68 (19)	289 (81)	**0.003**
Cardiac arrest b	10 (3.3)	293 (96.7)	5 (4.3)	39 (95.7)	15 (4.2)	342 (95.8)	**0.043**
30-day mortality	8 (2.6)	295 (97.4)	2(3.9)	52 (96.1)	10 (2.8)	347 (97.2)	0.66

Abbreviations: Acute Limb. Isch., acute limb ischemia; Anticoag. Compl., anticoagulation complication; ARR, absolute risk reduction; CT, full aortic computed tomography; ECMO, extracorporeal membrane oxygenation; Iliac Fem. Diss., iliac femoral dissection; NNT, number needed to treat; Operation (any cause), operation for any cause in association with aortic valve replacement; Other Neur. Compl., other neurological complication; Perioperative MI, perioperative myocardial infarction; Post. Stroke (72h), postoperative stroke within 72 hours; Cont. Coma (>24h), continuous postoperative coma for more than 24 hours; Prol. Ventilation, prolonged ventilation; Pulm. Embolism, pulmonary embolism.

a Any causes for reinterventions included bleeding/tamponade, pericardial effusion, valve dysfunction, other cardiac causes, or noncardiac causes associated to surgery such as hematoma evacuation, sternal stabilization, or treatment of sternal infection.

b Cardiac arrest may be triggered by postoperative arrhythmias, myocardial ischemia, bleeding/tamponade, electrolyte imbalances and other postoperative complications such as infection, embolism, or severe inflammatory response.

**Table 4:** Complications in patients aged ≥75 (current transcatheter aortic valve implantation recipients) in comparison to younger patients

In-hospital complications	CT ( *n* = 97)		No CT ( *n* = 14)		Total ( *n* = 111)		*p* -Value
	Yes, *n* (%)	No, *n* (%)	Yes, *n* (%)	No, *n* (%)	Yes, *n* (%)	No, *n* (%)	
Patients aged ≥ 75	24 (24.7)	73 (75.3)	8 (57.1)	6 (42.9)	32 (28.8)	79 (71.2)	0.012
	**CT (** ***n*** ** = 208)**		**No CT (** ***n*** ** = 40)**		**Total (** ***n*** ** = 248)**		
	**Yes,** ***n*** **(%)**	**No,** ***n*** **(%)**	**Yes,** ***n*** **(%)**	**No,** ***n*** **(%)**	**Yes,** ***n*** **(%)**	**No,** ***n*** **(%)**	
Patients aged ≤ 75	29 (13.9)	179 (86.1)	9 (22.5)	31 (77.5)	38 (15.3)	210 (84.7)	0.169

Abbreviation: CT, full aortic computed tomography.

### Survival Analysis

**Fig. 1:**
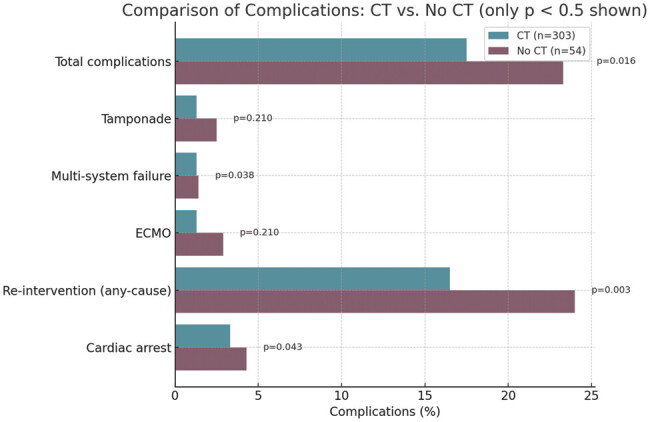
In-hospital complications in SAVR with versus without CT. Only complications with *p* < 0.5 are displayed. CT, computed tomography; ECMO, extracorporeal membrane oxygenation; SAVR, surgical aortic valve replacement. Source: © Liliane Zillner via Canva.com; Created with ChatGPT-5.

A tendency toward longer survival with presurgical CT was observed ( Table 5 , Fig. 1 ). Patients that experienced complications lived significantly shorter than patients without, emphasizing that preventing complications with preoperative CT might be crucial (total survival, *p* = 0.002, survival from surgery, *p* < 0.001).

**Table 5:** Survival analysis

Patients	CT ( *n* = 303)	No CT ( *n* = 54)	Total ( *n* = 357)	*p* -Value
Survival since surgery	3,257 (3100–3,414)	3,206 (2,830–3,583)	3,250 (3,105–3,395)	0.73
**Patients**	Complications ( *n* = 70)	No Complications ( *n* = 287)	Total ( *n* = 357)	
Survival since surgery	2,389 (1,994–2,785)	3,459 (3,317–3,601)	3,250 (3,105–3,395)	<0.001

Abbreviations: CT, full aortic computed tomography; Complications, In-hospital complications.

Notes: Time spans are calculated until the 2024. Data are calculated as median (95% confidence interval) for not normal distributed data.

### Impact of Computed Tomography on Surgical Strategy

In an extension of this analysis, an additional 213 SAVR patients who underwent AVR with full preoperative CT guidance were included, focusing solely on the influence within the surgical journey for these patients. This brought the extended total CT-informed study cohort to 518 patients. The majority of these patients ( *n* = 465/89.8%) underwent biological AVR, whereas 53 patients (10.2%) received mechanical AVR. Overall, 38 operations (7.3%) were redo procedures.

Severe calcification of the ascending aorta was identified via CT in 39 patients, representing a 4.2% incidence rate within the total study population. Upon reviewing CT findings, the surgeon opted for an alternative cannulation approach in five patients (1%), and 24 patients (4.8%) were transitioned from surgical AVR to transapical TAVI (TA-TAVI), with none experiencing a perioperative stroke.

In an additional analysis, we evaluated all instances where preoperative CT imaging prompted a change in surgical strategy, leading to a switch to transapical TAVI, accounting for 4.5% ( *n* = 24) of the CT cohort ( *n* = 532). None of the patients who underwent this switch experienced a stroke or transient ischemic attack.

## Discussion

The most significant finding of this study was the reduction in total in-hospital complication rates in the cohort that underwent a full preoperative aortic CT scan (CT cohort) (17%), compared with the patients who did not receive a CT (non-CT cohort) (32%; *p* < 0.016). Patients monitored with preoperative CT demonstrated a tendency toward improved survival, although this difference was not statistically significant.

Preoperative CT scans were significantly associated with the reduction of cardiac arrest, multisystemic failure, and reinterventions for any cause. The causes of reinterventions included bleeding (tamponade), pericardial effusions, valve dysfunctions, other cardiac complications, and noncardiac issues related to surgery, such as hematoma evacuation, sternal stabilization, and treatment of sternal infections. Preoperative CTs may have improved pre- and perisurgical management, potentially reducing the need for additional reinterventions. To further support our findings, we observed a consistent trend toward lower complication rates, regardless of the access route, as minimally invasive approaches are generally associated with fewer noncardiac complications. Surprisingly, no significant trend toward lower stroke rates was observed in CT patients, in contrast to larger studies focusing on CABG. 7 This discrepancy may be explained by the presence of a calcified aortic valve in SAVR, which is associated with approximately a 3-fold higher risk of stroke, as well as by the limited size of our study population. 19 Crucially, when complications did occur, both total lifespan and surgery-to-death lifespan were significantly reduced. This finding highlights that complications contribute to decreased survival, underscoring the importance of CT-enhanced surgical planning and execution.

Despite the 2021 ESC guidelines do only recommend echocardiography prior to SAVR, additional CT can provide relevant information serving the surgeon and the heart team to personalize and improve the surgical planning, conduction, or chosen intervention (SAVR or TAVI). 5 6 While current scientific literature offers insights into the use of preoperative CT, most studies have concentrated on redo procedures. They often examine diverse patient groups, leading to conclusions that are challenging to interpret. 20 Lee et al. reported that noncontrast CT was associated with lower rates of strokes and mortality and may be superior to epiaortic ultrasound for influencing surgical planning and decision-making. However, their study group was quite small, focusing only on a high-risk population ( *n* = 114). Additionally, the assessment included general cardiac interventions, not just SAVR, contributing to a potentially diverse study group. 21 A meta-analysis by Harder et al. suggested a reduction in stroke rates in 4,052 patients. 8 Moreover, while research on CT use prior to cardiac surgery primarily presents data on stroke prevention in CABG and general cardiac surgery, there is a lack of focus on populations exclusively undergoing SAVR. 7 22 23

The rising use of CT in our center reflects its growing clinical acceptance. To minimize confounding from temporal practice changes, we restricted the cohort to patients treated before 2015, when CT had become nearly universal in our clinic. Current evidence may indicate that preoperative CT scans reduce stroke rates and mortality in primary cardiac surgery, yet it has not been widely adopted. 8 Further studies, such as ours, are essential to support and demonstrate the potentially reduced complications and enhanced safety afforded by presurgical full aortic CT.

### Comparison to Transcatheter Aortic Valve Implantation Patients (Patients Aged ≥ 75)

Our study suggests that for patients aged 75 and older, who are nowadays common candidates for TAVI, the use of preoperative CT significantly reduces in-hospital complications. This underscores CT's potential importance in improving outcomes for this demographic group. Considering the yet unknown durability of TAVI, and the average life expectancy in European women at birth climbing up to > 85 years (Spain: 86.2 years, France: 85.5 years, Italy: 85.1), the findings of a 2023 meta-analysis of 8,698 patients acquire additional relevance. 24 25 This analysis did not find marked differences in outcomes between SAVR and TAVI among high-risk patients. These insights highlight the possibility that, with careful preoperative planning using CT, SAVR could remain a valid option for older individuals in the context of increasing life expectancies. As our findings demonstrate reduced complication rates in both elderly and younger patient cohorts, we encourage surgeons to consider preoperative CT for all patient groups.

### Surgical Planning and Neurological Complications

As known from other studies, routine preoperative CT can lower stroke rates in CABG or reoperative cardiac surgery patients. 7 26 27 Thus, we further investigated on surgical planning and neurological complications in an extended analysis of a total of 528 CT patients. CT-enhanced surgical planning in SAVR could indeed influence pivotal surgical steps like the cannulation strategy (five patients, 1%) or even altered the whole treatment approach by preferred TAVI (24 patients, 4.8%). Thus, preoperative CT led to fundamental changes in treating AS in at least 5.6% (29 patients) of this study cohort. Remarkably, none of these 29 patients experienced a periprocedural stroke, a devastating complication, with association to increased morbidity and mortality. 28 29

Despite the improvement in SAVR, periprocedural stroke is still a feared complication with a stroke risk of 1.3 to 2.6% in a worldwide population (meta-analysis: 3517 cases from 1985 to 2013). 29 30 31 Moreover, a perioperative stroke risk of 1.4% was assessed in a low–intermediate risk population (meta-analysis: 25,415 cases in 2019). 32 In comparison, at our department, the incident of perioperative strokes in a high-risk population was remarkably low (1.2%), as shown by this analysis of 518 patients. This could result from detailed preoperative planning by the heart team, which included detailed imaging of the ascending aorta, where the cross-clamping takes place. Severe calcifications can be identified early in the planning process, and a possible switch to a TAVI could be considered. We might assume that the presurgical conducted CT for every patient could add to our comparably low stroke rates.

## Limitations

The current study is retrospective in nature and therefore does not eliminate a selection or sampling bias, among other usual limitations of retrospective analysis. Furthermore, outcome data were available for the analyzed cohort, but previous patients could only be analyzed for switch of therapy (extended analysis). Data are not only retrospective, but also represent a rather old surgical cohort, prior to the rise of TAVR. This is also related to the fact that our department switched to 100% preoperative CT nowadays due to our excellent clinical experience, which make comparisons to non-CT patients in the recent patient population not feasible. Finally, the challenge of assessing CTs beneficial effects on SAVR planning, conduction, and personalization requires further studies.

## Conclusion

Our findings indicate that preoperative full aortic CT correlates with a reduced incidence of in-hospital complications following SAVR. Therefore, we advocate for the integration of CT into standard preoperative protocols as a noninvasive diagnostic tool. Employing CT facilitates the identification of patients with atheromatous aortic disease, thus allowing for tailored surgical planning. This personalized approach can inform the optimization of surgical strategies or, where appropriate, a transition to TAVI.

**Fig. 2:**
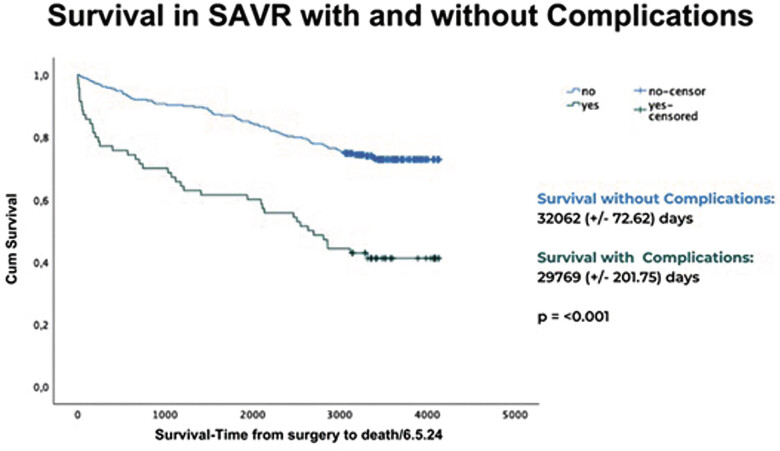
Surgery-to-death survival in SAVR patients with and without complications. Cum Survival, cumulative survival; SAVR, surgical aortic valve replacement.
